# Microwave assisted sol–gel approach for Zr doped TiO_2_ as a benign photocatalyst for bismark brown red dye pollutant†

**DOI:** 10.1039/d3ra00328k

**Published:** 2023-03-15

**Authors:** Gorli Divya, G. Jaishree, T. Sivarao, K. V. Divya Lakshmi

**Affiliations:** a Dept of Chemistry, Andhra University Visakhapatnam 530003 India sivaraoau@gmail.com; b Bio Enviro Chemical Solutions Visakhapatnam 530003 India

## Abstract

A microwave supported sol–gel approach was developed in this study to fabricate Zr-doped TiO_2_ mesoporous nanostructures for efficient photocatalytic activity on bismark brown red (BBR) dye under visible light illumination. Sophisticated analytical techniques such as X-ray diffraction (XRD), X-ray photoelectron spectroscopy (XPS), high resolution transmission electron microscopy (HRTEM), field emission scanning electron microscopy (FESEM) with energy dispersive X-ray spectroscopy (EDX), X-ray fluorescence analysis (XRF), Fourier transform infrared (FT-IR), ultraviolet-visible diffuse reflectance (UV-vis-DRS) spectroscopy and Brunauer–Emmet–Teller (BET) surface area analyses were used to obtain their structural, electrical: optical and spectroscopic characteristics. The analysis results revealed that the developed nanostructures exhibited strong broad absorption in the visible region with good adsorption capacity and thus enhanced photocatalytic performance. The average crystallite size was found to be 12.5 nm (UTO), 6.4 nm (ZT_4_), and 4.7 nm (ZT_4_M_4_) respectively. The nanocatalysts (ZT_4_M_4_) showed a decrease in bandgap and particle size with an increase in the surface area of the Zr-TiO_2_ nanoparticles (119 m^2^ g^−1^). In comparison to previous studies on the photocatalytic degradation of BBR dye under visible light irradiation employing Ni–S co-doped (110 min), Cu-doped TiO2 (75 min), *etc.*, ZT_4_M_4_ exhibited a remarkable degradation rate of 99% in 50 minutes. This may be due to the hydroxyl radicals being the principle reactive species responsible for the BBR dye oxidative degradation. The present study showed that ZT_4_M_4_ was found to be the best photocatalyst for the BBR dye degradation under the optimal conditions.

## Abstract 2

A microwave supported sol–gel approach was developed in this study to fabricate Zr-doped TiO_2_ mesoporous nanostructures for efficient photocatalytic activity on bismark brown red (BBR) dye under visible light illumination.

## Graphical abstract

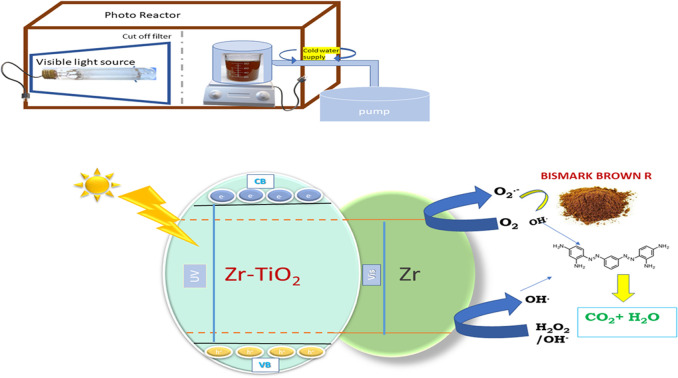

## 1 Introduction

When dated back, the heating impact of microwaves was discovered by accident while working on radar applications.^1,2^ The use of microwave irradiation has progressed as a simple, rapid, and mild synthesis route for producing a wide variety of nanomaterials with perfect control over their morphology and size.^3,4^ In addition, it has enabled the rapid implementation of novel reactions with broad applications in multiple disciplines of the chemical sciences, such as solid-state chemistry, nanomaterial synthesis,^5^ nanotechnology,^6^ and organic synthesis.^7^ It is an *in situ* method^8^ of conversion of energy that provides the advantage of consistent dielectric heating without the consequences of thermal gradients,^9^ as the heat is generated internally within the particles rather than from external sources.^10^ It uses fewer chemicals and is therefore considered an environmentally friendly process.^11^ Due to their lower activation energy and greater diffusion rates, microwaves can minimise processing temperature while creating small particle size, controllable morphology, purity, and homogeneous microstructure.^12,13^

Thus, microwave-assisted synthesis is one of the prospective tools to ameliorate the quality of life in the field of nanoscience and technology, resulting in a substantial advancement in the large-scale synthesis of numerous functional metal oxide nanomaterials with novel structures and properties.^14,15^ Also, because microwave radiation is so important and involved in making metal oxide nanomaterials, it can be used to make doped semiconductor nanomaterials that are active in visible light.

TiO_2_ is one of the best semiconductors due to its chemical and biological inertness, high performance under UV light irradiation and long-term photostability.^16^ Based on the above the application of microwaves has advanced as a simple, quick, and mild synthetic route for creating a wide range of nanomaterials with precise control over their morphology and size^3^ merits of TiO_2_, Ding *et al.* produced TiO_2_ nanocrystals having anatase phase with identical shape and size.^17^ Cui *et al.* created TiO_2_ nanotubes using a hydrothermal process assisted by microwaves.^18^ Simonsen *et al.* used the microwave-assisted sol–gel technique to make TiO_2_ films, which they then used to study how OH groups affect TiO_2_'s ability to act as a photocatalyst.^19^ Their research showed that this method makes thin films of TiO_2_ that are very uniform and do not need a high-temperature heat treatment to crystallise. Unfortunately, TiO_2_'s high photo-generated charge carrier recombination rate and large band gap (3.2 eV) limit it from being employed as a visible-light photocatalyst.^20^ To overcome the aforementioned drawbacks and improve TiO_2_'s quantum efficiency in the visible range, the electron–hole (e^−^/h^+^) recombination and band gap must be lowered by doping TiO_2_ with non-metal or metal ions. To ameliorate the drawbacks of TiO_2_, researchers reported the synthesis of metal doped and non-metal doped TiO_2_ with the assistance of microwave radiation.

In the framework of the present study, microwave assisted sol gel technique was used to fabricate zirconium-doped TiO_2_. As zirconium is biologically inert, nontoxic and possesses the same valence shell state, valence shell structure and comparable ionic radii as titanium (Zr-0.72 Å, Ti-0.68 Å), which can facilitate the substitutional doping of Ti^4+^ with Zr^4+^, which results in reducing the band gap and high efficiency in the segregation of photogenerated charge carriers.^21,22^ Their porosity results in a enhanced absorption, high surface area, and rapid transfer of contaminants.^23^ Numerous reports describe the fabrication of Zr-doped TiO_2_ with enhanced photocatalytic activity by using high calcination temperatures.^24,25^ Therefore, it is necessary to design a method for generating nanoparticles at low temperatures. For the Zr-doped TiO_2_ synthesis, we opted a microwave-irradiation method, which has various advantages over traditional heating, such as high thermal homogeneity, quick and selective heating, which will assist in lowering calcination temperatures.^26^ Various methodologies are employed to characterise the synthesised catalysts.

The degradation of BBR dye was used to evaluate the photocatalytic efficacy of the synthesised nanocatalysts. BBR dye is a negative dye that is persistent in the environment and causes carcinogenicity. It is primarily employed in the leather and textile industries as a colouring agent.^26,27^ The ecosystem is in grave danger as a result of the discharge of these dyes as washouts into the aquatic environment.

## 2 Experimental

### 2.1 Required chemicals and reagents

Tetra butyl *ortho* titanate Ti(*O*Bu)_4_ and zirconium nitrate Zr(NO_3_)_2_ are the main precursors of titanium and zirconium, and they are used to synthesize Zr-doped and undoped (UTO) TiO_2_ catalysts. The compounds obtained were AR-Grade E. Merck chemicals (Germany). From High media India the Bismarck brown red an anionic textile dye (pollutant) is obtained. In the reaction procedure, E-Merck (India) nitric acid and Hayman ethanol are utilised. All of the aforementioned compounds were employed without purification, and using deionised water their solutions were freshly prepared.

### 2.2 Synthesis of Zr-doped TiO_2_

The synthesis process involves two steps:

#### 2.2.1 Step 1: synthesis using the sol–gel technique

Sol–gel synthesis was employed in the first step to synthesize Zr-doped TiO_2_ nanoparticles with varying dopant weight percentages (ZT_1_ (0.00882 g), ZT_2_ (0.01765 g), ZT_3_ (0.02646 g), ZT_4_ (0.0353 g) and ZT_5_ (0.0441 g)). In first beaker, ethanol (40 mL) was mixed with *n*-butyl *ortho* titanate (20 mL) and stirred for 10 minutes (Solution I). The mixture was then acidified with nitric acid (3.2 mL) by stirring continuously. Later in second beaker, Solution II was prepared by taking the necessary amount of zirconium, 40 mL of ethanol, and H_2_O (7.2 mL) are taken and stirred for 30 minutes. Then, to the Solution I, Solution II is added gently while stirring rapidly. After repeating the procedure, the mixture is stirred continuously for an additional two hours until the formation of clear sol. The resultant sol is left in the dark for 48 hours at ambient temperature to form a gel. After drying the gel in an oven at 80 °C, the catalyst was ground with a pestle and mortar and calcined for five hours at 450 °C in a muffle furnace. The catalyst was calcinated and crushed into a powder form. The photocatalysts were identified with the designations ZT_1_, ZT_2_, ZT_3_, ZT_4_, and ZT_5_.

#### 2.2.2 Step 2: synthesis using sol–gel technique assisted by microwaves

Based on the analytical results of UV-vis DRS, XRD, and the photocatalytic ability of the catalysts, ZT_4_ was determined to be the best catalyst due to its reduced band gap, low crystallite size, and superior photocatalytic capability. Therefore, ZT_4_ was selected, and the same technique was followed until formation of sol, after which the mixture was divided into five equal portions. Then the sol has to set for 48 hours at room temperature in the dark to form gel. Each sample is then microwave-irradiated at varying wattages (180 W, 360 W, 540 W, 720 W, 900 W). The microwave power levels and irradiation time were represented in Table 1. Here the role of microwave irradiation is employed not only for drying, but it also reduces reaction time and has various other benefits such as less nucleation, which leads to smaller particle size, high reproducibility, and uniform morphology. The prepared catalysts were ground and calcined in muffle furnace at 350 °C for five hours, and then ground again. The labels for the prepared samples were ZT_4_M_1_, ZT_4_M_2_, ZT_4_M_3_, ZT_4_M_4_, and ZT_4_M_5_. The same procedure was employed to develop undoped (UTO) TiO_2_. The names allocated to the catalysts were reported in Table 2.

**Table:** Microwave power levels and irradiation time

Power level	Output	Time
Low	180 W	20 min
Medium low	360 W	20 min
Medium	540 W	15 min
Medium high	720 W	15 min
High	900 W	10 min

Names assigned to different weight percentages of Zr-doped TiO_2_ catalysts

**Table:** 

S. no.	Dopant weight percentages Zr/wt%	Name assigned to catalyst
1	0.25	ZT_1_
2	0.50	ZT_2_
3	0.75	ZT_3_
4	1.00	ZT_4_
5	1.25	ZT_5_

**Table:** 

S. no.	At different watts	Name assigned to catalyst
Microwave assisted sol gel synthesized catalyst		
1	180 W	ZT_4_M_1_
2	360 W	ZT_4_M_2_
3	540 W	ZT_4_M_3_
4	720 W	ZT_4_M_4_
5	900 W	ZT_4_M_5_

### 2.3 Sophisticated instrumental techniques are employed for the characterization of the catalyst

The instrument Shimadzu 3600 UV-vis DRS NIR spectrophotometer was used to determine the band gap and absorption edges of all the co-doped catalysts and undoped TiO_2_. In order to measure the UVDRS values between 200 nm and 800 nm, the reference standard BaSO_4_ was loaded by mixing with nano samples as pellet in the quartz cell. The instrumental technique PXRD (model-Ultima IV Rigaku) equipped with an anode Cu Kα at source *λ* = 1.5406 nm was used to identify the crystalline phase of Ba/Cu co-doped titanium dioxide and undoped TiO_2_ nanomaterials. From the FWHM results, the average crystallite sizes for all the samples measured using the Debye–Scherer equation. By using PHI quantum ESCA microprobe XPS system was used to determine the oxidation states and elemental composition of the catalysts. These spectra were captured by using X-ray radiation of 1253.6 eV with Al Kα 250 W at 16 mA current, 12.5 kV voltage. The particle size, shape, and SAED pattern of the nanoparticles were obtained from the High Resolution-Transmission Electron Microscope (model: Jeol/JEM 2100) at 200 kV equipped with a LaB6 electron gun having point resolution of 0.23 nm and a lattice resolution of 0.14 nm. The Field Emission Gun-Scanning Electron Microscope (FEG-SEM) of model JEOL JSM 7600F with an Energy Dispersive X-ray spectrophotometer (EDX) operated at 20 kV and was used to evaluate the photocatalyst's elemental composition and surface morphology. The X-ray Fluorescence (XRF) with X-ray tube 50 kV max, 1 mA with Rh target (XGT 5200, Horiba, Japan) was also used to determine the elemental composition. The pore size, surface area, and pore volume of the photocatalyst were calculated using the Brunauer–Emmett–Teller (BET) surface area analyzer (model: Gemini VII 2390 series micro metrics). The FTIR spectra were recorded using FTIR spectrometer (Bruker, Germany model: 3000 Hyperion Microscope with Vertex 80 FTIR system) in the range of 400–4000 cm^−1^. The photoluminescence spectral analysis was done by using a setup of both 150 V PMT and 2.5 nm slit (Horiba Jobin Fluoro Max-4). A Shimadzu 1601 UV-visible spectrophotometer was used for investigation of the extent and rate of degradation of dye. Elico's digital pH meter (model: IIIE, EI) was used for monitoring and adjusting the pH of reaction mixture during the degradation process. The microwave power source utilized for synthesis is LG model no. MC2846SL of microwave power 1350 W and frequency 2450 MHz with RF output of 900 W.

### 2.4 Framework for photocatalytic activity of the catalyst-degradation of BBR dye

The photocatalytic effectiveness of the produced catalysts was determined by observing the breakdown of BBR dye in the presence of visible light. To optimise the performance of catalysts, reaction parameters were investigated. In the experimental setup for photocatalytic degradation, a high pressure 400 W (35 000 lumen) metal halide lamp (Osram, India) is utilised as the visible radiation source the distance between the beaker and radiation source with the UV-blocking filter was maintained at 20 centimetres (Oriel, No. 51472). The reaction beaker has a cold water supply circulating around it to filter off infrared radiation and to maintain the reaction at ambient temperature throughout the entire procedure. Prior to irradiation, the pH of the solution was adjusted by adding either 0.1 N HCl or 0.1 N NaOH.^28,29^ In order to achieve equilibrium between dye and catalyst, 100 mL of BBR dye solution was combined with a certain amount of catalyst at a particular pH in a reaction beaker and swirled continuously for 30 minutes in the absence of visible light. After exposing the reaction mixture to visible light for degradation, aliquots of 5 mL are periodically removed using a Millipore syringe. Using a UV-vis spectrophotometer, the variation in the concentration of dye is monitored by measuring its absorbance at (max 459 nm). The following formula was used to calculate the percentage of dye degradation (BBR).Percentage of degradation = *A*_0_ − *A*_*t*_/*A*_0_ × 100*A*_0_ corresponds to absorbance of the dye solution prior to light exposure. At corresponds to initial absorbance of the dye solution after exposure to light at time *t*.

The reaction parameters were optimised by varying conditions such as concentration of dopant, effect of pH, catalyst concentration, and initial concentration of dye.

## 3 Results and discussion

### 3.1 XRD analysis

**Fig. 1:**
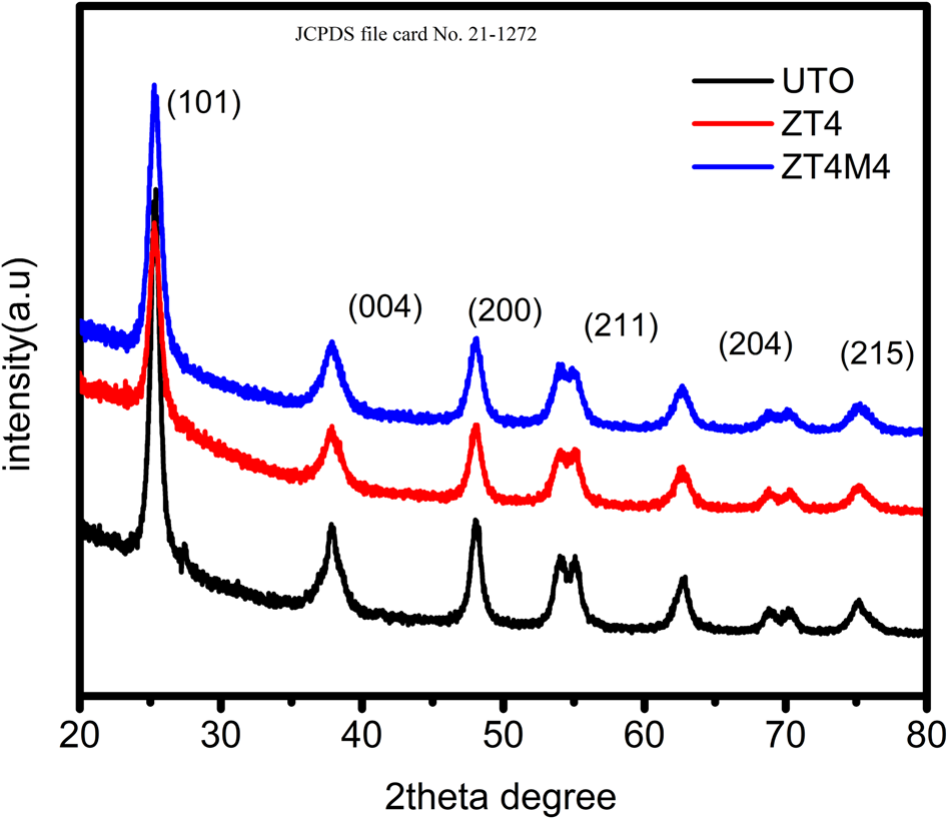
XRD pattern of undoped, ZT_4_, and ZT_4_M_4_ catalysts.

As shown in Fig. 1, the powder XRD patterns depicted the phase and structure of doped and undoped TiO_2_ samples. The 2*θ* peak values were determined to be 25.3°, 37.8°, 48.2°, 54.8°, 55.2°, 62.7°, 69.8°, 70.5°, and 75.2°, indicating a sign of the presence of an anatase. These results demonstrated that the anatase phase of TiO_2_ is not influenced by the presence of dopants. The absence of a peak at 2*θ* = 27.8° indicates that Zr^4+^ ion doping into TiO_2_ lattice has no effect on the structure of the material. Zr^4+^ ions replace Ti^4+^ ions since Zr^4+^ has a ionic radius close to that of Ti^4+^ (0.720 Å). This could be evidence that no additional peaks for ZrO_2_ and ZrTiO_4_ were found.^30^ During synthesis, the replacement of Ti^4+^ ions with Zr^4+^ ions inhibit crystallite growth. The Debye–Scherrer equation determines the average size of crystallites.^31^*D* = *kλ*/*β* cos *θ*where *β* is the FWHM in radians, *θ* is the Braggs angle, and ‘*λ*’ is the wavelength of X-ray (*A*). The average crystallite size was found to be of 12.5 nm (UTO), 6.4 nm (ZT_4_), and 4.7 nm (ZT_4_M_4_), respectively.

### 3.2 XPS

**Fig. 2:**
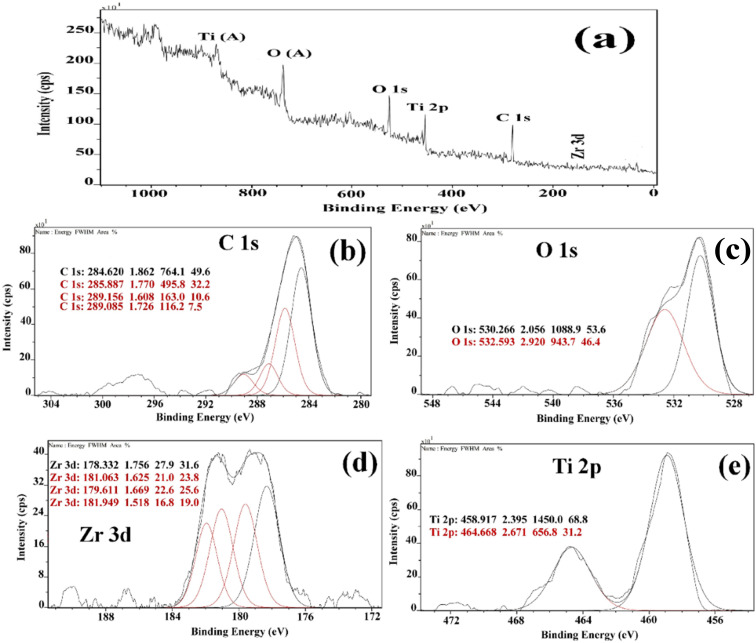
(a) XPS survey spectrum of Zr doped TiO_2_ and high resolution spectrum of (b) c 2p (c) O1s (d) Zr 3d (e) Ti 2p Respectively.

The oxidation states and chemical composition of elements in the ZT_4_M_4_ were investigated using XPS. The complete survey spectrum of ZT_4_M_4_ elemental composition is depicted in Fig. 2a, which includes titanium, oxygen, zirconium, and standard carbon. This spectrum represented the existence of dopant elements in the catalyst. By evaluating the magnification spectra of the elements, Fig. 2e shows that the binding energies of 464.11 eV and 458.003 eV corresponds to doublet peaks at Ti 2p_1/2_ and Ti 2p_3/2_, respectively. The presence of the Ti^4+^ ion is indicated by the 6.10 eV splitting energy between these two peaks. Ti^4+^ in undoped TiO_2_ has binding energies of Ti 2p_3/2_ and Ti 2p_1/2_ of 464.6 eV and 459.9 eV, respectively with a peak energy splitting difference of 5.7 eV, which is lower than doped TiO_2_.^32^ This minor shift is due to the incorporation of Zr^4+^ into the TiO_2_ lattice. The O1s spectrum in Fig. 2c has doublet peaks, lattice oxygen in Ti–O bonds contributes to the prominent peak at 530.266 eV, while weakly physically adsorbed oxygen species on the surface (OH and O^2−^ groups), account for the smaller peak at 532.593 eV.^32^ The Zr^4+^ oxidation state is responsible for the absorption of the Zr 3d_5/2_ and Zr 3d_3/2_ peaks, which have binding energies of 178.250 eV and 181.250 eV, respectively (Fig. 2d).^33^ The spectra thus affirmed the incorporation of zirconium into the lattice of TiO_2_ and also the presence of a strong interaction between the dopants and the TiO_2_ lattice.

### 3.3 Morphology study

**Fig. 3:**
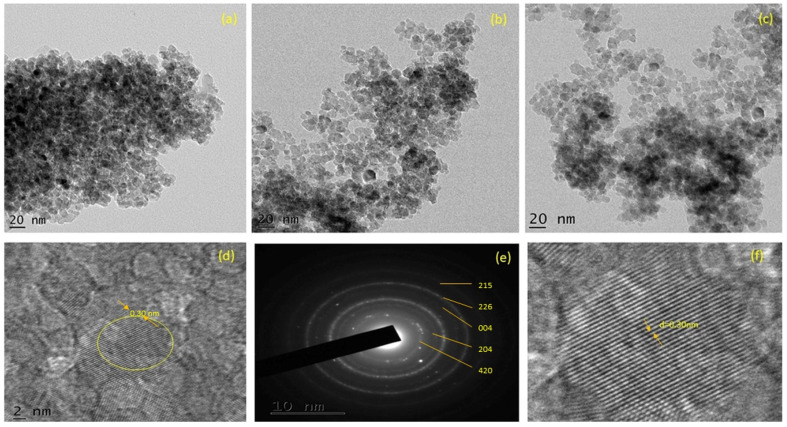
TEM micrograph of (a) UTO, (b) ZT_4_, (c) ZT_4_M_4_, (d) HRTEM image of ZT_4_M_4_, (e) SAED pattern and (f) lattice fringes of ZT_4_M_4_.

Fig. 3a–c depict TEM images of ZT_4_M_4_, ZT_4_, and UTO nanocatalysts, in addition to their particle size distributions. ZT_4_ and ZT_4_M_4_ particles (Fig. 3b and c) exhibit significantly less agglomeration and smaller particles than TiO_2_ particles (Fig. 3a) with pseudospherical shape. The HRTEM image reveals that Zr-TiO_2_ contains nanocrystals with anatase phases that are randomly oriented. It was determined that the particles interplanar spacing (0.30 nm) corresponded to the anatase plane (*d*-spacing). Fig. 3d displays selected region electron diffraction patterns in addition to HRTEM (SAED). The existence of a crystalline anatase phase is also pointed by concentric rings. Using Gaussian fitting method, histograms of particle size distribution were generated to reveal the average particle size.^34^ According to the data, the average particle size of ZT_4_M_4_ is smaller than ZT_4_, which is smaller than UTO, which measures 13, 6.5, and 5.3 nm.

**Fig. 4:**
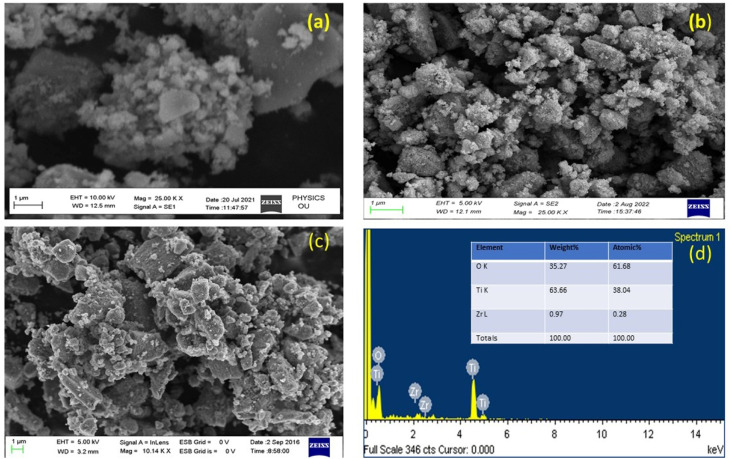
FESEM image of (a) undoped TiO_2_, (b) ZT_4_, (c) ZT_4_M_4_, and (d) EDX spectrum of ZT_4_M_4_.

FESEM and EDX analyses were used to investigate the morphological properties and composition of prepared UTO, ZT_4_, and ZT_4_M_4_ catalysts, and the corresponding images are shown in Fig. 4a–d. The SEM images of UTO ZT_4_ and ZT_4_M_4_ show a spherical shape with rough morphology, indicating effective Zr doping of TiO_2_. The chemical composition was determined by EDX in the range of 0 to 14 keV binding energy area, given in Fig. 4d. The spectral peaks at 4.52, 4.96, and 0.54 keV K correspond to the components Ti and O of TiO_2_. The presence of Zr was also indicated by two diffraction peaks that were seen at 2.1 and 2.5 keV. The quantitative results (Fig. 4d inset) confirmed the presence of 0.97 wt% Zr relative to Ti (9.87 wt% is taken as 100%), which coincides with the quantity of doped Zr (1.00 wt%). The SEM analysis of catalysts at different power levels was represented in ESI S1.†

### 3.4 X-ray fluorescence analysis

X-ray fluorescence analysis is a widely accepted effective method for determining the qualitative and quantitative composition of a material by irradiating the sample with high-energy X-photons and observing the outgoing X-ray fluorescence.^15^ This approach provides for the accurate identification of chemical elements with atomic numbers ranging from 16 (sulphur) to 92 (uranium). The experimental results indicated that the wt% of the doped element were almost coincides with the actual amount doped into the TiO_2_ lattice given in Table 3. The spectral data corresponding to all these samples were shown as ESI (S2).†

**Table:** The chemical composition of catalyst samples, by the X-ray fluorescence analysis

Sample	Titanium (wt%)	Zirconium (wt%)
ZT_1_	99.77	0.23
ZT_2_	99.56	0.44
ZT_3_	99.28	0.72
ZT_4_	99.04	0.96
ZT_5_	98.81	1.19

### 3.5 FTIR study

**Fig. 5:**
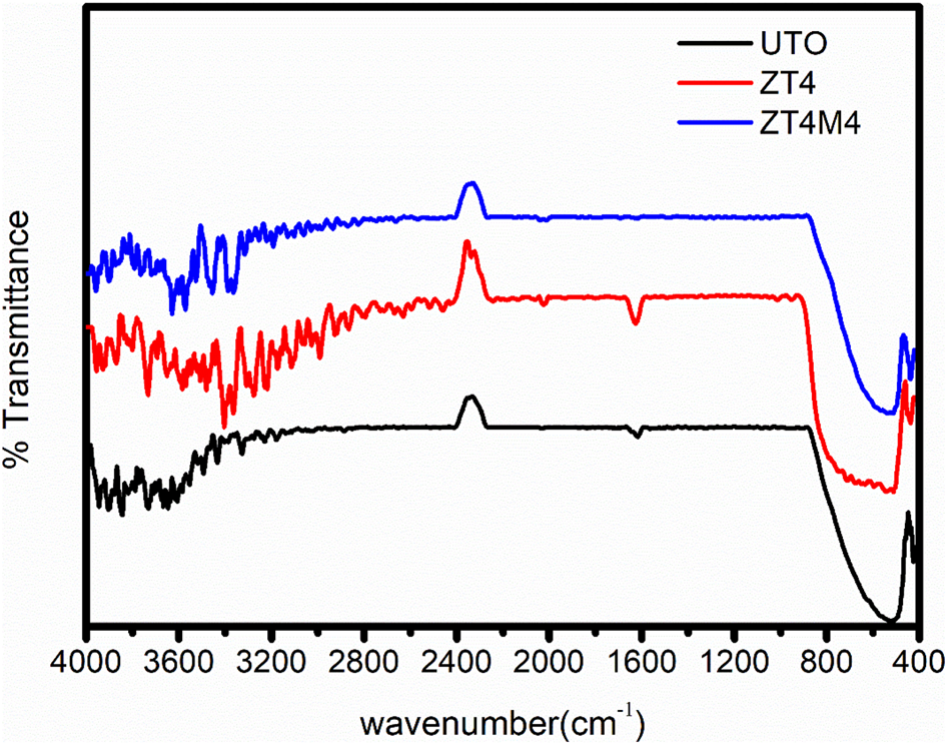
FTIR spectra of UTO, ZT_4_, and ZT_4_M_4_.

The FTIR spectra of ZT_4_, ZT_4_M_4_, and UTO nanoparticles are represented in Fig. 5. The catalysts that were synthesised exhibited a broad absorption band in the vicinity of 3660–2600 cm^−1^; this band corresponded to the surface hydroxyl group's (Ti–OH) stretching vibration. Additionally, the samples exhibited an absorption peak at 1631 cm^−1^; this peak was ascribed to the (H–OH) bending vibration of water molecules.^35^ Characteristic absorbance peaks at 549 indicate the stretching vibrations of Ti–O–Ti in UTO which where it is moved towards lower wavenumber of 490 cm^−1^ (range 400–900 cm^−1^). This change is a result of the successful substitutional doping of Zr^4+^ into the lattice of TiO_2_ in place of Ti^4+^ Due to the development of the Zr–O network, the adsorbed –OH stretching band has shifted.^36^ No characteristic band is observed at 470 cm^−1^, which indicates the absence of ZrO_2_. Due to the development of the Zr–O network, the adsorbed –OH stretching band has shifted. The peak around 619–835 cm^−1^ is attributed to stretching and bending vibrations of the Zr–O–Ti bond,^37^ which clearly says that Zr is doped into the TiO_2_ lattice.

### 3.6 BET analysis

**Fig. 6:**
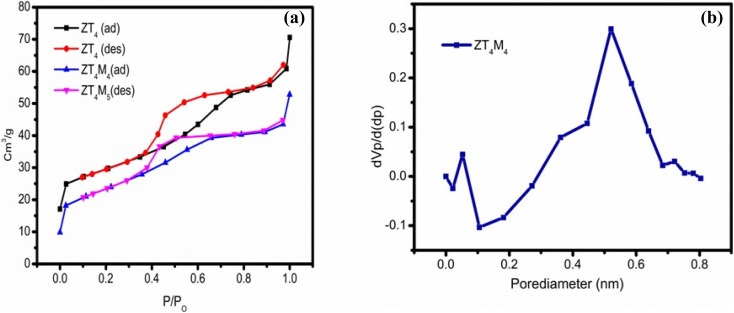
BET (a) N_2_ adsorption–desorption isotherms and (b) BJH pore size distribution curves.

The pore size, surface area, and pore volume analysis of the UTO, ZT_4_ and ZT_4_M_4_ photocatalytic nanomaterials were determined by BET analysis. The N_2_ adsorption desorption measurements of the samples show type-IV isotherms with H2 hysteresis loops which are represented in Fig. 6a.^38^ The corresponding pore size distributions are estimated and using the BJH method, which is based on isothermal adsorption and are given Fig. 6b. ZT_4_M_4_ was observed to have a higher surface area (119 m^2^ g^−1^) over ZT_4_ (90 m^2^ g^−1^), which is higher than UTO. According to the data, the particles have high surface area and are mesoporous. In the Table 4 are the appropriate values for pore size, pore volume, and surface area. ZT_4_M_4_ synthesised with the aid of microwaves has a greater adsorbent surface area than ZT_4_, indicating that microwave-assisted synthesis increases the nanomaterials' surface area.

**Table:** The results of crystallite size (XRD), band gap (UV-vis-DRS) & BET surface area

S. no.	Catalyst code no.	Crystallite size (nm)	Band gap energy (eV)	BET surface area analysis		
				Surface area (m^2^ g^−1^)	Pore volume (cm^3^ g^−1^)	Pore size (nm)
1	UTO	12.5	3.07	61	0.21	4.2
2	ZT_4_	6.4	2.68	90	0.43	4.8
3	ZT_4_M_4_	4.7	2.66	119	0.52	5.8

### 3.7 UV-vis DRS spectral analysis

**Fig. 7:**
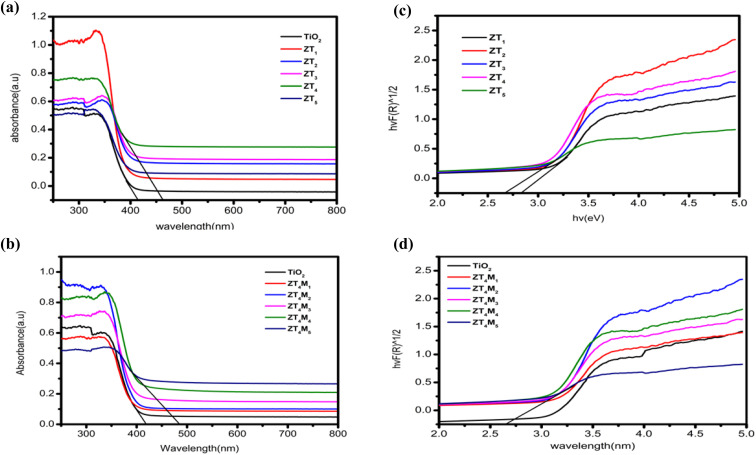
UV-vis DRS (a, b) the DRS spectra and (c, d) plots of transformed square root of Kubelka–Munk functions of UTO, ZT and ZT_4_M samples.

UV-vis DRS was employed to study the photoabsorbance characteristics of UTO, ZT_4_, and ZT_4_M_4_ samples in the wavelength range of 200–800 nm. The undoped TiO_2_ shows its absorption at 300–400 nm wavelength range. Due to the metal–ligand charge transfer between O^2−^ (valence band) and Ti^4+^ (conduction band) and reflects the most light in the visible range of 400 to 800 nm. According to Fig. 7a, Zr^4+^ doping shifts the absorption edge (red shift) towards higher wavelengths, *i.e.* the visible light region. This is due to TiO_2_ being swapped by Zr^4+^ ions, resulting in intermediate energy levels or surface trap states.^39^ These smaller band gaps and red shifts were probably generated by defects induced by smaller doses of Zr^4+^ in the TiO_2_ lattice or by enlarged crystal domains.^40^ Using the Tauc plot method, and Kubelka–Munk formalism the band gap of the catalysts was determined using reflectance spectra [*F*(*R*)]. From the figure, the band gap energies of the intercepting target will approximate the band gap energies of the samples. The predicted band gap values for UTO and ZT (ZT_1_–ZT_5_) samples are between 3.07–2.68 eV.

Among them, ZT_4_ samples show a lower band gap of 2.68 eV, which was further fabricated using the microwave-assisted approach and the band gaps of as-synthesized ZT_4_M (ZT_4_M_1_–ZT_4_M_5_) samples were determined and found to be within 2.8 and 2.66 eV. The band gap of ZT_4_M_4_ was the smallest at 2.66 eV. The results demonstrated that Zr-doping increased absorption intensity, causing the samples' photo response to shift towards the visible region. The Table 2 is provided with band gap of each sample for comparison.

The characterisation data of XRD, BET, UV-vis DRS were represented in Table 4.

## 4 Assessment of the photocatalytic activity of the catalyst

**Fig. 8:**
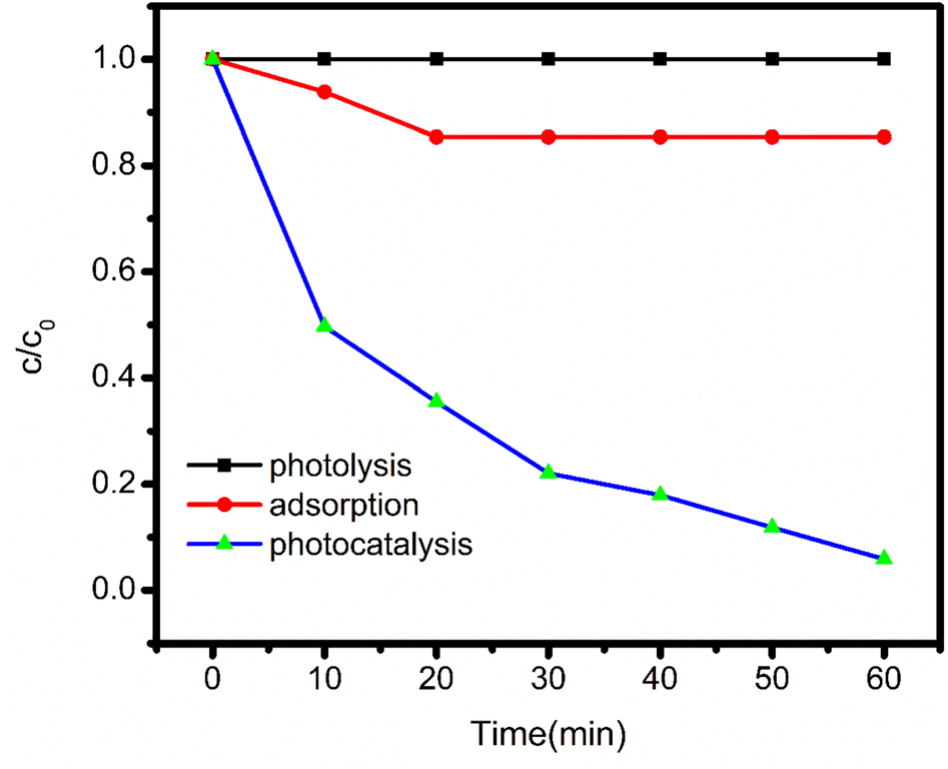
Shows the effect of adsorption, photolysis and photocatalysis of BBR dye.

Under visible light irradiation, experiments were conducted to investigate the photodegradability and adsorption capability of BBR dye with as synthesised catalyst. Before starting with the aforesaid procedure, preliminary experiments were undertaken to evaluate the interdependency between dye, catalyst, and visible light; the results are depicted in Fig. 8.

### 4.1 Photolysis

In a 150 mL Pyrex glass container, 100 mL of a 5 mg L^−1^ BBR dye solution was poured and exposed to the light for 1 hour. 5 mL samples were collected at different intervals of time, the reaction mixture's absorbance was determined at 459 nm. There was no visible distinction in the dye's absorption. The deterioration of BBR dye is implied to be unaffected by light in the figure by a straight line that is parallel to the *X*-axis.

### 4.2 Adsorption

In the reaction vessel the solution containing 5 mg L^−1^ dye with a concentration of 50 mg L^−1^ catalyst at pH 3 was taken. The reaction mixture was kept in dark, continuously stirred for 60 min and the absorbance as measured. A modest decrease in dye absorbance was observed, indicating dye molecule adsorption on the charged catalyst's surface.

### 4.3 Photocatalysis

The above-mentioned reactants (catalyst and dye) were placed in another reaction vessel and subjected to the visible light radiation under continuous stirring for 60 minutes. At different time intervals, the absorbance was measured by collecting 5 mL aliquots of the dye solution. The absorbance of the dye showed a drastic change caused due to activation of the catalyst. These outcomes indicate that both catalyst and the light were essential for the degradation of the dye.

The efficiency of the catalyst is also reliant on a variety of operating reaction parameters, including initial BBR dye concentration, dopant concentration, pH influence, and catalyst dosage. The influence of these parameters was investigated to attain the optimal conditions for catalyst's high photocatalytic activity.

### 4.4 Influence of dopant loadings on the photocatalytic activity of TiO_2_

**Fig. 9:**
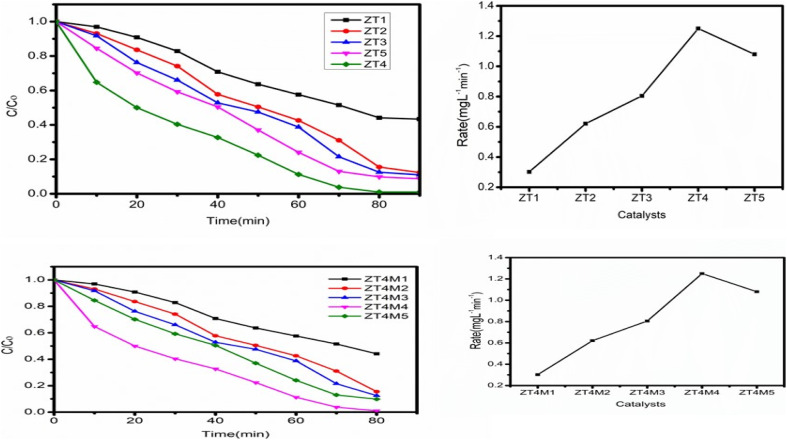
Shows the effect of dopant concentration on degradation of BBR dye. Ccatalyst dosage 0.05 g L^−1^, solution pH 3, and initial dye concentration 5 mg L^−1^.

Experiments were conducted with dopant concentrations ranging between 0.25–1.25 wt% to determine the effect of dopant loadings on visible light-induced degradation of BBR dye catalysts. Keeping other parameters constant, like solution pH 3, catalyst dosage of 0.05 g L^−1^, and initial dye concentration of 5 mg L^−1^. Fig. 9 demonstrates that, relative to UTO, the photocatalytic performance of all catalysts (ZT_1_–ZT_5_) showed better performance than UTO. Among all, ZT_4_ had the best efficiency of any doped sample due to the greatest reduction in bandgap, which enables higher quanta absorption per particle in visible light radiation, hence enhancing photocatalytic activity.

In addition to light absorption, the effectiveness of the catalyst is dependent on the surface area of the particle, which facilitates dye adsorption on the catalyst's surface. Consequently, the scissoring of particle size with microwave irradiation is an ecologically benign procedure that decreases particle size. On this basis, the ZT_4_ catalyst was synthesised utilising microwaves, and the photocatalytic performance of the produced catalysts (ZT_4_M_1_, ZT_4_M_2_, ZT_4_M_3,_ ZT_4_M_4_, and ZT_4_M_5_) was evaluated using the method described before. Fig. 9 demonstrates that ZT_4_M_4_ possesses excellent photocatalytic activity. We have therefore chosen ZT_4_M_4_ as the most effective catalyst for the breakdown of BBR dye.

### 4.5 Role of pH on the photocatalytic activity of TiO_2_

**Fig. 10:**
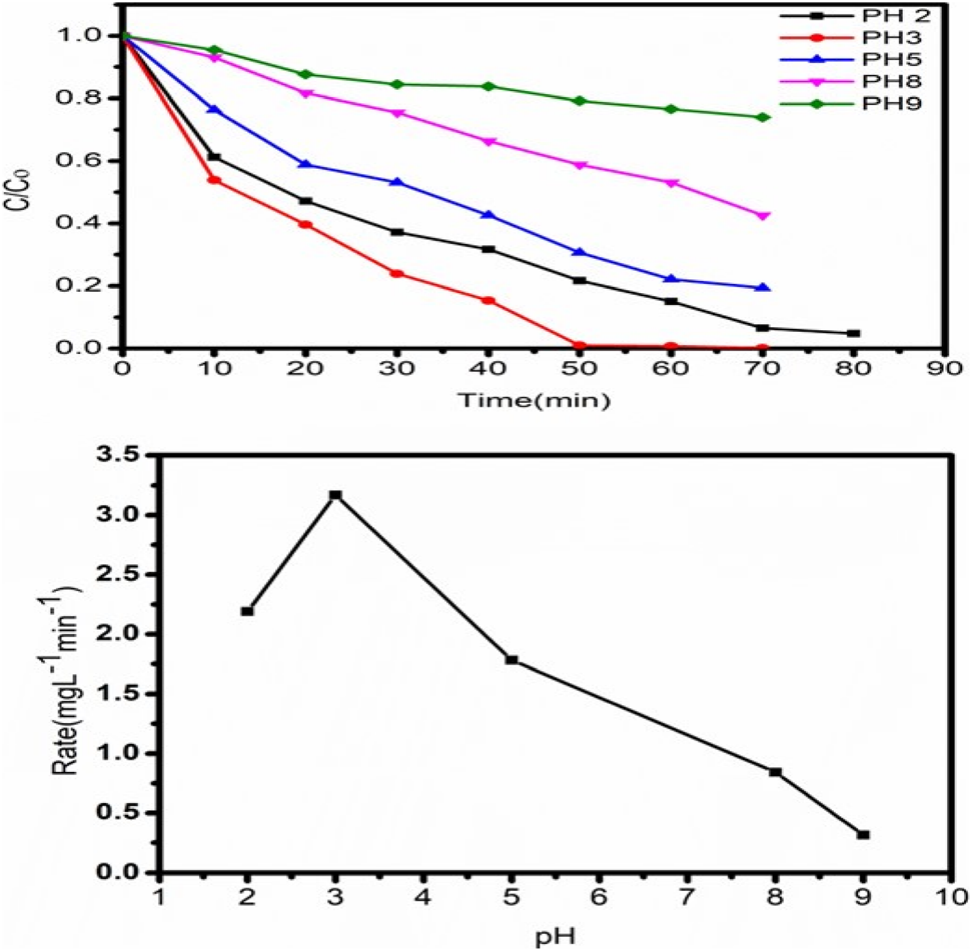
Shows the impact of solution pH on the degradation of BBR dye.

The initial pH of the solution is also crucial for the catalyst's photocatalytic efficiency changes in pH can alter the surface property of the catalyst, thereby affecting the adsorption of the target compounds. Therefore, the pH effect on the photocatalytic degradation and degree of adsorption of dye utilising (ZT_4_M_4_) nanomaterial was examined by altering pH 2, 3, 5, 8, and 9 while keeping the other parameters constant: catalyst dosage at 0.05 g L^−1^ and 5 mg L^−1^ of initial dye concentration. The results are displayed in Fig. 10. The adsorption of negatively charged dye molecules enhanced by Electrostatic interaction under acidic circumstances because the surface titanol group (Ti–OH) is protonated (Ti–OH_2_^+^), hence increasing the surface area. Consequently, the rate of breakdown of adsorbed dye molecules has risen. Due to a decrease in the positive charge on the catalysts surface, the concentration of H^+^ ions at pH 5 decreases.^41^TiOH + H^+^ → TiOH_2_^+^, pH < 6.25

Titanol (Ti–OH) is converted to TiO^−^ by the removal of a water molecule as the pH increases. This is due to the fact that at basic pH, the charge on the catalyst's surface becomes negative. Therefore, it may not be feasible to adsorb the negatively charged dye molecules, and the degradation rate is low.^42^ According to the data, the ideal pH for dye degradation is 3, as the catalyst's positively charged surface increases dye adsorption.TiOH → TiO^−^ + H^+^, pH > 6.25

### 4.6 Efficacy of the catalyst as determined by its concentration

**Fig. 11:**
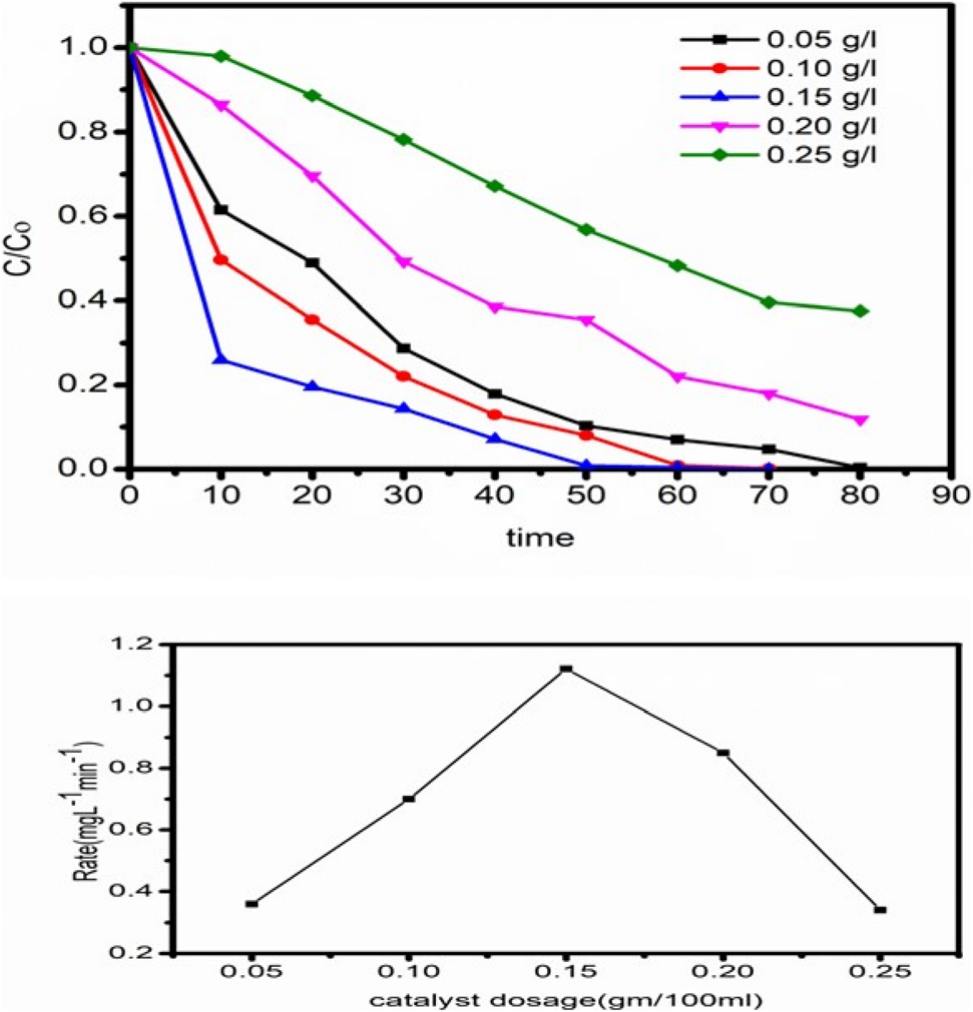
Shows the effect of surfactant concentration on degradation of BBR dye.

To determine the impact of catalyst concentration (ZT_4_M_4_) on the photocatalytic BBR dye degradation. Experiments were conducted with catalyst concentrations ranging from 0.05 mg L^−1^ to 0.25 mg L^−1^, while pH 3 and initial dye concentration of 5 mg L^−1^ remained unchanged Fig. 11 depicts the experimental results demonstrating that the degradation rate increases, as the amount of catalyst increases up to 0.15 mg L^−1^, then decreases gradually. After attaining the ideal concentration of catalyst, degradation slows as a result of an increase in turbidity of the solution and non-availability of dye molecules.^43^

### 4.7 The significant effect of the initial dye concentration

Examining the impact of initial dye concentration on the photocatalytic activity of ZT_4_M_4_ nanomaterial. Experiments were conducted by increasing the dye concentration from 5 mg L^−1^ to 20 mg L^−1^, with a constant dose of 0.15 mg L^−1^ catalyst at a pH of 3. The outcomes are shown in the figure. The degradation rate enhances with increase in dye concentration up to 10 mg L^−1^, at which point it decreases. Inspite of an increase in the dye molecules concentration, the degradation rate reduces due to the absence of active catalyst particles. In other terms, the decrease in dye degradation rate is well explained by the blanket effect,^44^ which states that once the first layer of dye molecules is adsorbed on the surface of the catalyst, it may not be possible to adsorb a second layer. As a result of the blanket effect, the second layer's adsorption is delayed until the initial layer is totally degraded. Thus, increasing the dye concentration slows the rate of degradation.

The optimal conditions for effective (99%) degradation of BBR dye by ZT_4_M_4_ were 0.15 g L^−1^ of catalyst, 10 mg mL^−1^ of dye, and a pH of 3.

### 4.8 Scavenger tests to identify active species in ZT_4_M_4_ photocatalysis

Scavenger reagents were used to identify e^−^/h^+^, superoxide radical, and hydroxyl radical; EDTA, 1,4-benzoquinone, and coumarin reagents were used for the identification of above said reactive species.^45^ Under the impact of these chemicals, BBR dye degradation studies using visible light photocatalysis were conducted.

#### 4.8.1 Recognition of e^−^/h^+^

**Fig. 12:**
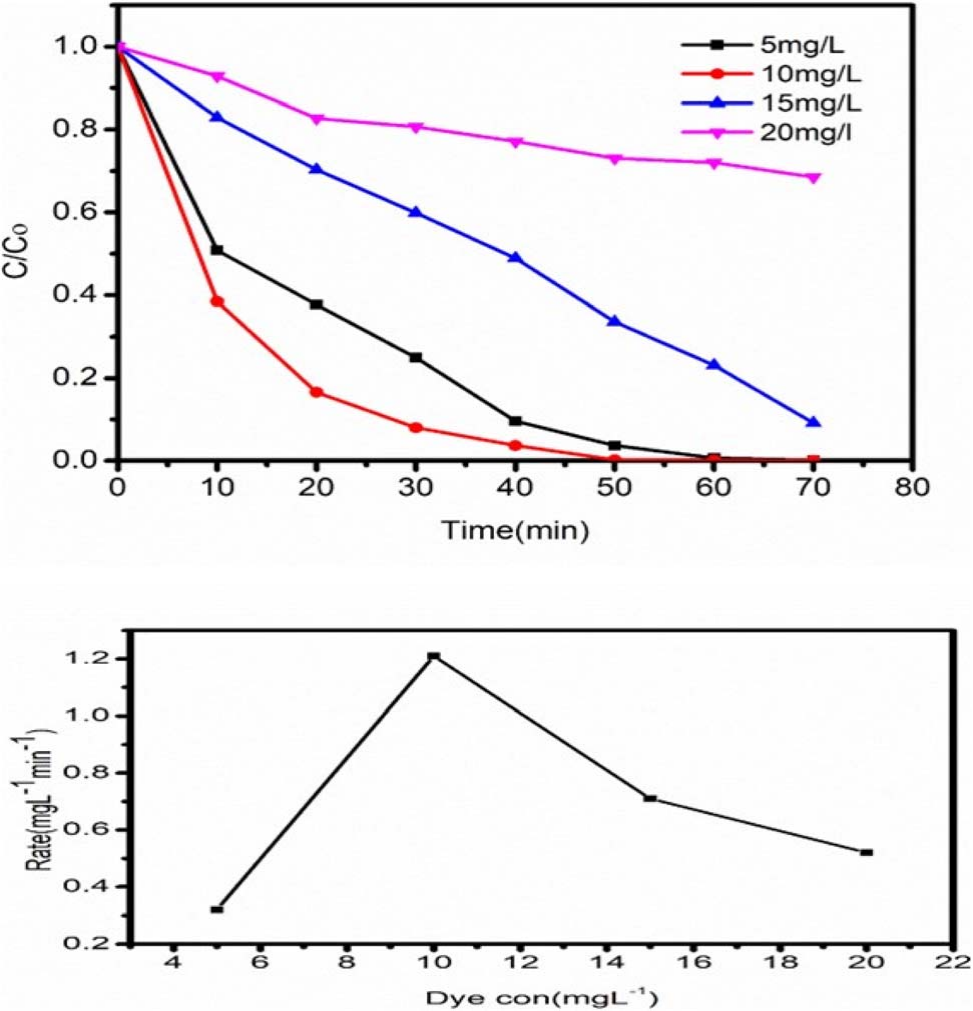
The effect of initial dye concentration on BBR dye degradation.

ZT_4_M_4_, 10 mg L^−1^ of dye, and 0.15 g L^−1^ of catalyst were added to a 150 mL pyrex glass beaker, and the pH level was maintained at 3. After exposing the reaction mixture to visible region for up to 5 minutes, 5 mL aliquots were obtained to measure the decrease in BBR dye absorbance. After extracting the aliquot, 1.0 mL of a 1 mM di-sodium EDTA salt solution was added. In the subsequent thirty minutes, samples were obtained every 5 min. The absorbance was measured, and the results are represented in the Fig. 12; it can be observed that the rate of degradation of BBR dye increased for the first 10 min after adding EDTA, but then slowed and remained constant at 15 minutes. This could be the result of EDTA's capacity to mask the activity of e^−^/h^+^, an essential site for dye degradation.

#### 4.8.2 Identification of superoxide radicals

**Fig. 13:**
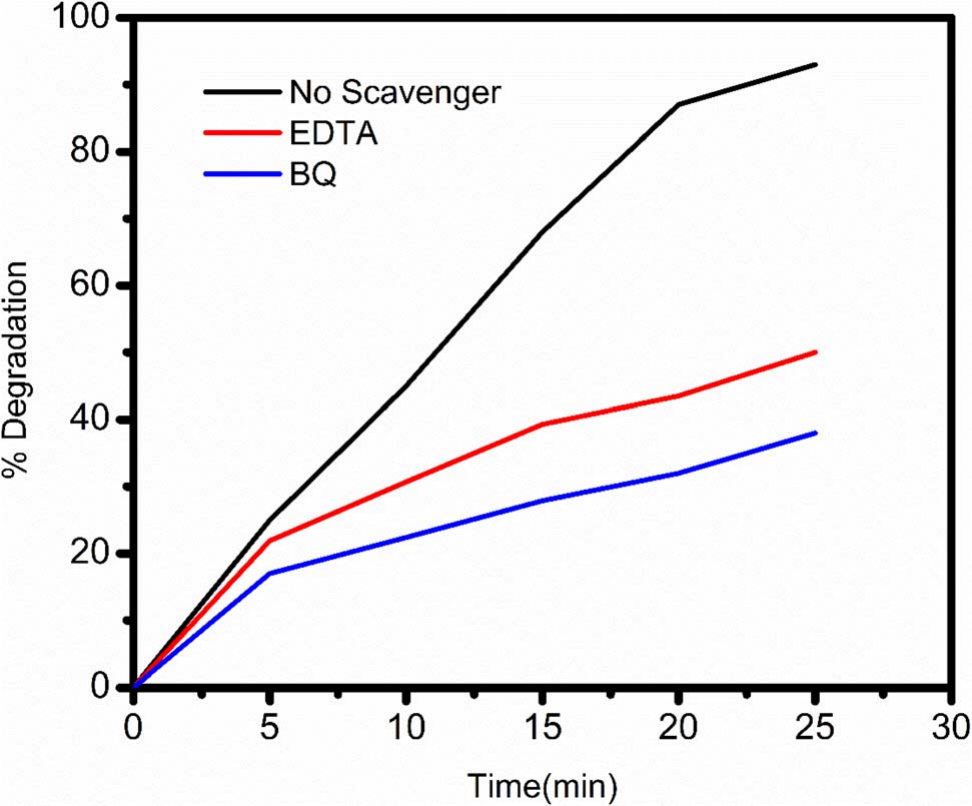
Effect of e^−^ hole and superoxide radicals (˙O_2_^−^) scavenger on BBR dye degradation.

In order to assess the production of O_2_˙^−^, 10 mg L^−1^ of dye and 0.15 g L^−1^ of catalyst were put into a different reaction vessel designated as ZT_4_M_4_, and the pH was maintained at 3. The mixture was exposed to visible light for 5 min and 1,4-benzoquinone (a superoxide radical scavenger) was added. For a duration of 30 min, 5 mL aliquots were collected every 5 minutes. As depicted in Fig. 13, the addition of 1,4-benzoquinone to the reaction mixture reduced the photocatalytic activity of BBR. This indicates that O_2_˙^−^ is a crucial intermediate in the resulting reaction that generates ˙OH.

#### 4.8.3 Photoluminescence

**Fig. 14:**
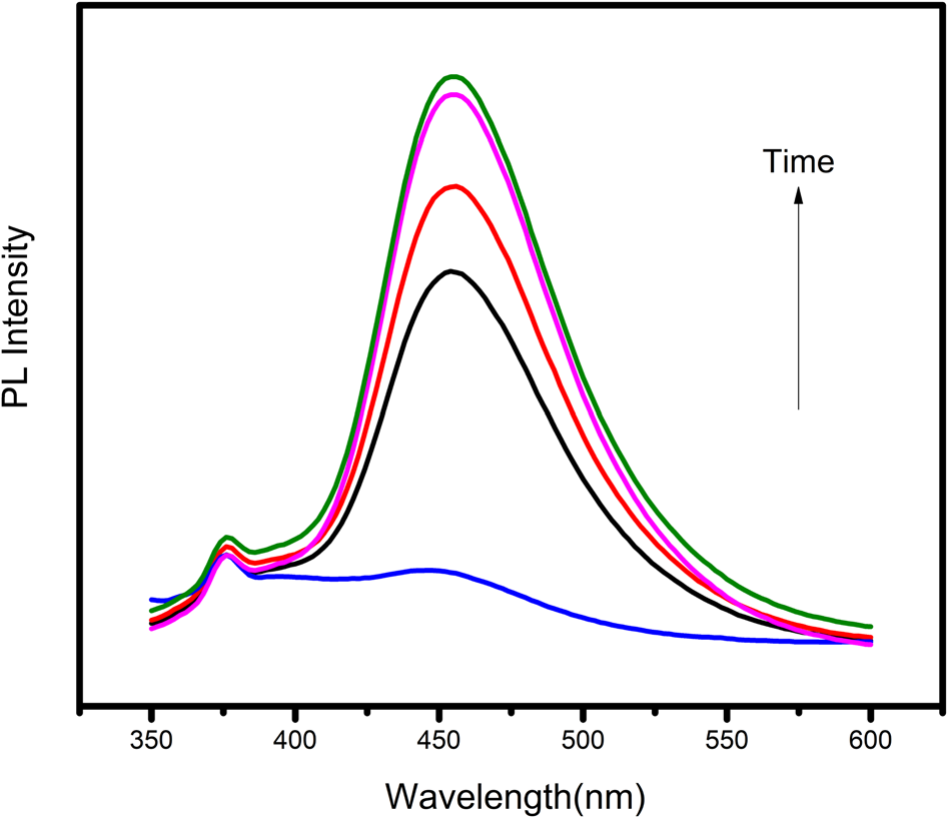
Photoluminescence spectra of ZT_4_M_4,_ catalyst dosage (0.15 g L^−1^), dye concentration (10 mg L^−1^), pH 3.

The hydroxyl radical is among the most important reactive species involved in degradation reactions and the oxidative degradation of pollutants. It is difficult to directly detect hydroxyl radicals because they are highly reactive and have a short lifetime. Using a fluorescent probe molecule, photoluminescence was used to determine the level of ˙OH radical production (coumarin). The coumarin probe interacts with an ˙OH radical to form 7-hydroxycoumarin during the reactions. The catalyst was mixed in a 10 mg L^−1^ acidic coumarin solution and exposed to visible light. The reaction solution was separated every 10 min, and the photoluminescent spectrum of the produced 7-hydroxy coumarin showed maximum absorption at 450 nm represented in Fig. 14. As the duration of irradiation increased, so did the spectrum's intensity. In addition, the results revealed that the synthetic sample ZT_4_M_4_ accelerated the synthesis of ˙OH. This is due to the ability of ZT_4_M_4_ catalyst particles to generate ˙OH when exposed to light.

### 4.9 Stability test

When considering the potential of a catalyst, its stability is a crucial factor to consider. Throughout the photocatalytic reactions, the catalyst must be durable and sustain high photoactivity for practical applications. In addition, the catalyst should be conveniently collected or recovered after each cycle of reaction. After being exposed to visible light, the catalyst's recyclability was evaluated. Each time, the photocatalyst degradation pattern was examined for approximately 50 minutes under ideal reaction conditions. After every 50 minutes of photocatalytic degradation, the catalyst was removed from the solution and rinsed with deionized water before being reused. Throughout this entire process, the optimal conditions for decomposition were always maintained. Fig. 16 depicts the slight reduction in photocatalytic activity of the ZT_4_M_4_ catalyst towards the dye over the period of four cycles. In acidic settings, however, BBR dye continued to degrade with high repeatability and robust photocatalytic activity. This phenomenon has an effect on the photoactivity of the catalyst due to the difficulty of cleaning its surface.

Later in order to further determine its stability of the catalyst after recycling process of degradation of BBR dye XRD and FEBBR analysis have been performed.

XRD was analysed to determine the crystalline nature and was found to be in anatase phase same as that of pure catalyst. The peaks were still evident in the recycled catalyst, showing that there were no much deformations in the catalyst even after it had been through four cycles.

**Fig. 15:**
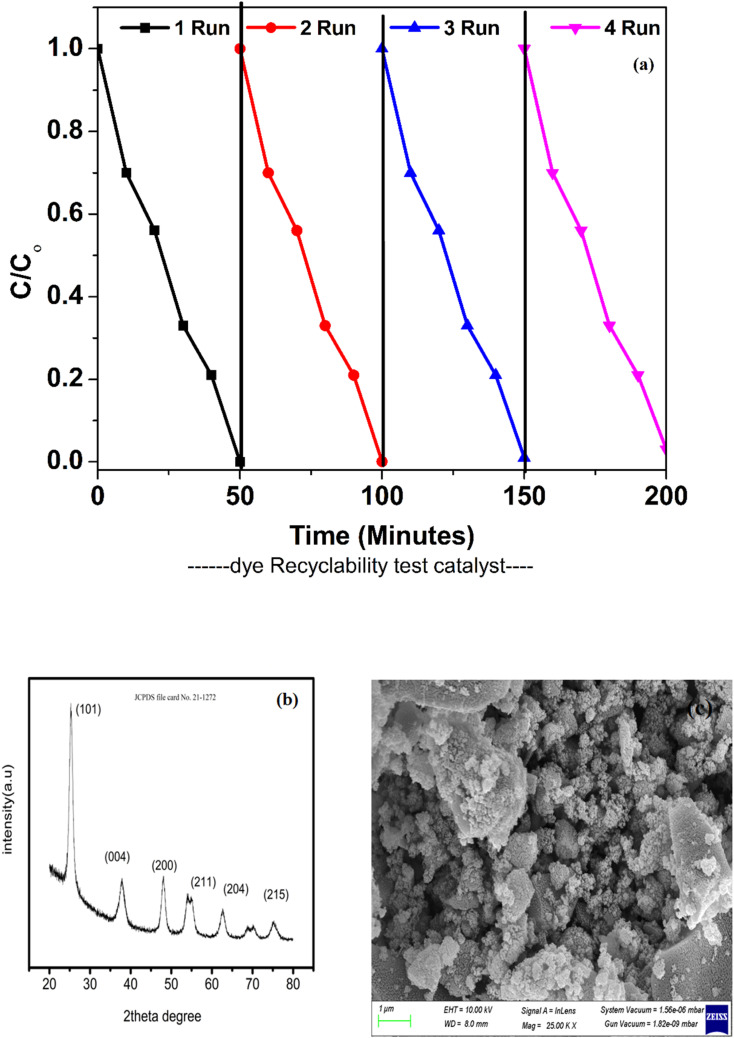
(a) Recyclability, (b) XRD analysis and (c) SEM analysis after recyclability.

FESEM analysis was used to analyse the surface morphology of the recycled catalyst. The analysis revealed that the surface was still uniform with a rough texture, with minimal change in morphology. The above results show that the catalyst retained its stability after recycling. The figures corresponding to recyclability test, XRD and SEM analysis were represented in Fig. 15.

### 4.10 A precise mechanism for ZT_4_M_4_'s photocatalytic activity

When visible light strikes Zr-TiO_2_, shifting of an electron from the valence band to the conduction band takes place, thereby creating a hole in the valence band.Zr-TiO_2_ + *hv* → Zr-TiO_2_ (VBh^+^) + Zr-TiO_2_ (CBe^−^)Zr-TiO_2_ (CBe^−^) + Zr^3+^ → Zr-TiO_2_ + Zr^2+^

The hydroxyl radical and H^+^ ion is produced when generated electron holes react with H_2_O or the surface hydroxyl group.Zr-TiO_2_ (VBh^+^) + H_2_O → Zr-TiO_2_ + OH^−^ + H^+^Zr-TiO_2_ (VBh^+^) + OH^−^ → Zr-TiO_2_ + ˙OH

In reaction with O_2_, the excited electrons in the conduction band generate super oxide radicals.Zr-TiO_2_ (CBe^−^) + O_2_ → Zr-TiO_2_TiO_2_ + O_2_^−^˙

This reaction produces a hydroperoxyl radical and a hydroxyl ion.Zr-TiO_2_ (CBe^−^) + O_2_^−^˙ + H_2_O → Zr-TiO_2_ + HO_2_˙ + OH^−^

Together with h^+^, the hydroperoxyl radicals form an intermediate. In turn, hydrogen peroxide oxidises the holes to produce hydroxyl radicals and hydroxyl ions.Zr-TiO_2_ (CBe^−^) + HO_2_˙ + H^+^ → Zr-TiO_2_ + H_2_O_2_Zr-TiO_2_ (CBe^−^) + H_2_O_2_ → Zr-TiO_2_ + OH˙ + OH^−^

These hydroxyl radicals attack and degrade the BBR dye, adsorbing it to the surface of the catalyst.OH(*hν*) + pollutant → degradation products

**Fig. 16:**
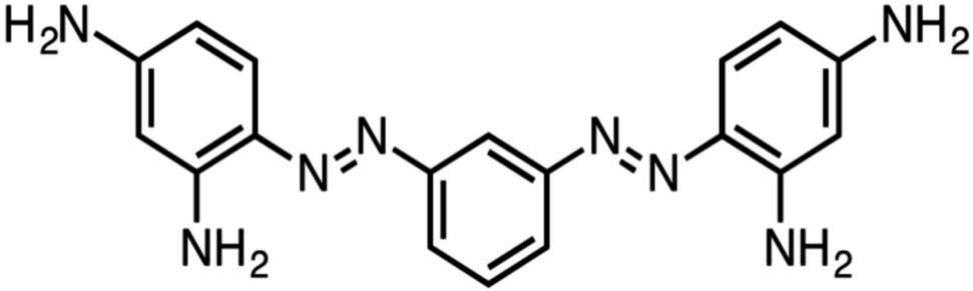
Structure of bismark brown red dye.

**Fig. 17:**
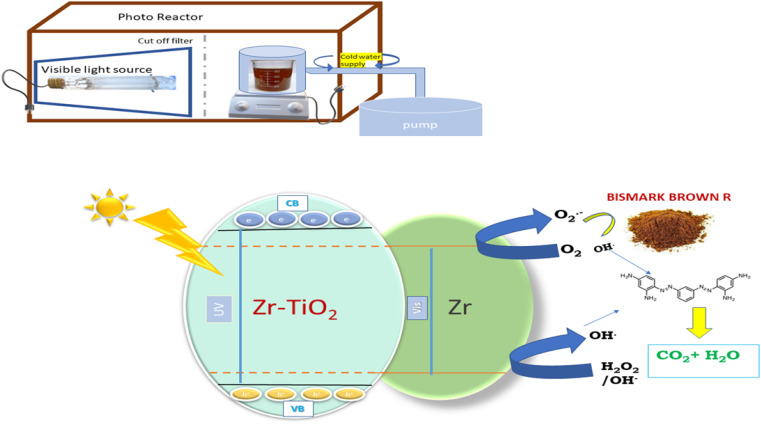
Schematic representation of photocatalytic degradation of BBR dye.

The structure of BBR dye and schematic representation of photocatalytic activity was given in Fig. 16 and 17.

The comparative study on degradation of BBR Dye was represented in Table 5.

**Table:** Comparative table for photocatalytic degradation of BBR

Nanomaterial nano catalyst	Dye pollutant	Degradation/%	Degradation time	Ref.
Ni–S co-doped	Bismark brown	99	110 min	20
Fe_0_/HP/UV	Bismark brown	73	60 min	46
Cr_2_O_3_–NiO	Bismark brown	97	60 min	47
Cu	Bismark brown	95.6	75 min	48
ZT_4_	Bismark brown	99	80 min	Present study
ZT_4_M_4_	Bismark brown	99	50 min	Present study

## 5 Conclusions

In this study, the synthesis of Zr-doped TiO_2_ using the microwave assisted sol gel technique is thoroughly described. The complete strategic method was devised to evaluate thoroughly the BBR dye degradation capability of as-produced Zr-doped TiO_2_ driven by visible light. ZT_4_M_4_ was found to be the most efficient of the as-synthesized catalysts, which can be attributed to its rough spherical morphology and lattice planes that coincide with the XRD, confirming the pure anatase form of the catalyst. ZT_4_M_4_ demonstrated the best performance in terms of reduced crystallite size (4.7 nm), band gap (2.66 eV), and surface area (119 m^2^ g^−1^). The TEM clearly displays the comparable particle size distribution of ZT_4_M_4_ without any agglomeration. The photodegradability of BBR dye, which reached 99% after 50 min of exposure to visible light, demonstrates the characterization results. The scavenger test demonstrates the presence of reactive species such as superoxide radicals (˙O_2_), e-holes, and hydroxyl radicals (˙OH).

## Conflicts of interest

Authors stated that no conflicts of interest.

## Supplementary Material

RA-013-D3RA00328K-s001
