# Supplementary material for: Microwave assisted sol–gel approach for Zr doped TiO2 as a benign photocatalyst for bismark brown red dye pollutant
Source: RSC Adv. 2023 Mar 15;13(13):8692–705. doi: 10.1039/d3ra00328k (PMC10015583; doi:10.1039/d3ra00328k)
Supplement: RA-013-D3RA00328K-s001 [file RA-013-D3RA00328K-s001.pdf]

Electronic Supplementary Material (ESI) for RSC Advances.
This journal is © The Royal Society of Chemistry 2023

Supplementary information

S1 FESEM images of ZT_4M at different microwave power levels. a) 180 W, b)360 W, c) 540 W, d) 720 W, and e) 900W

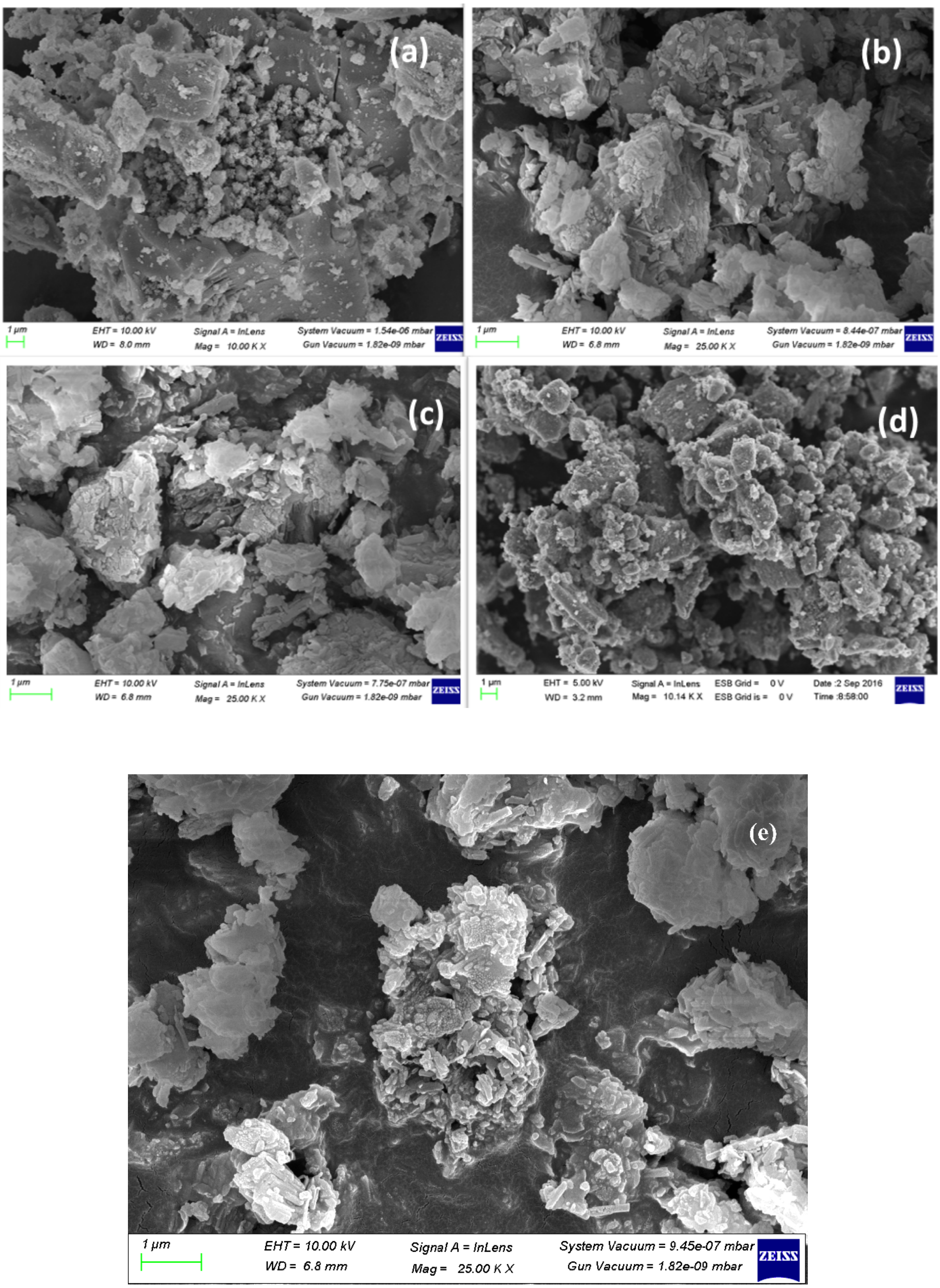

S2 XRF analysis a)ZT_1 b)ZT_2 c) ZT_3 d)ZT_4 d)ZT_4 e)ZT_5

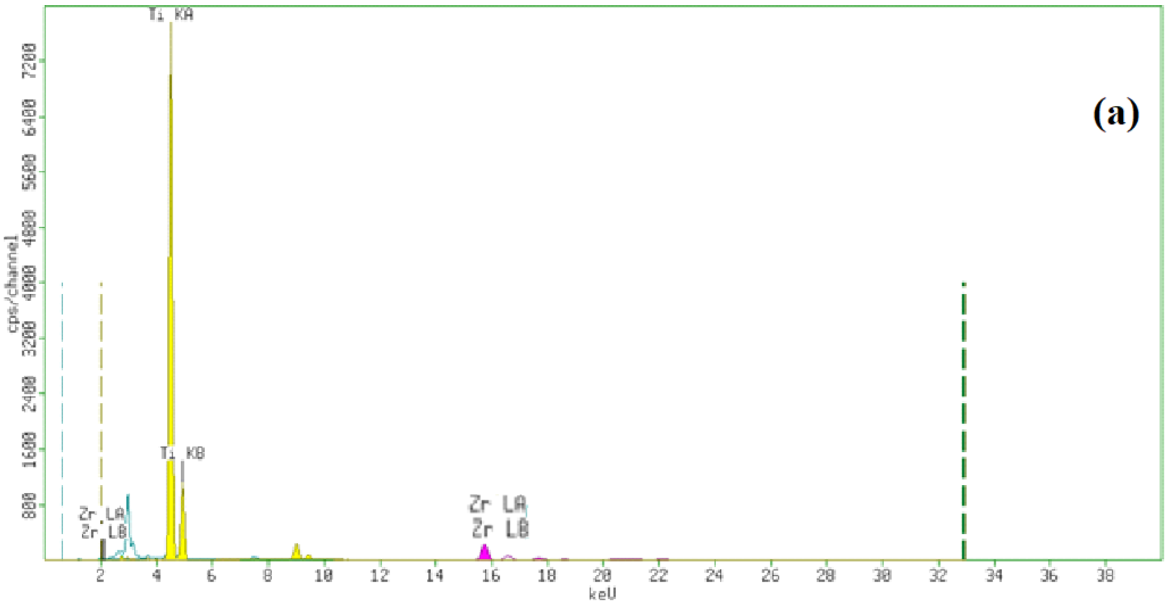

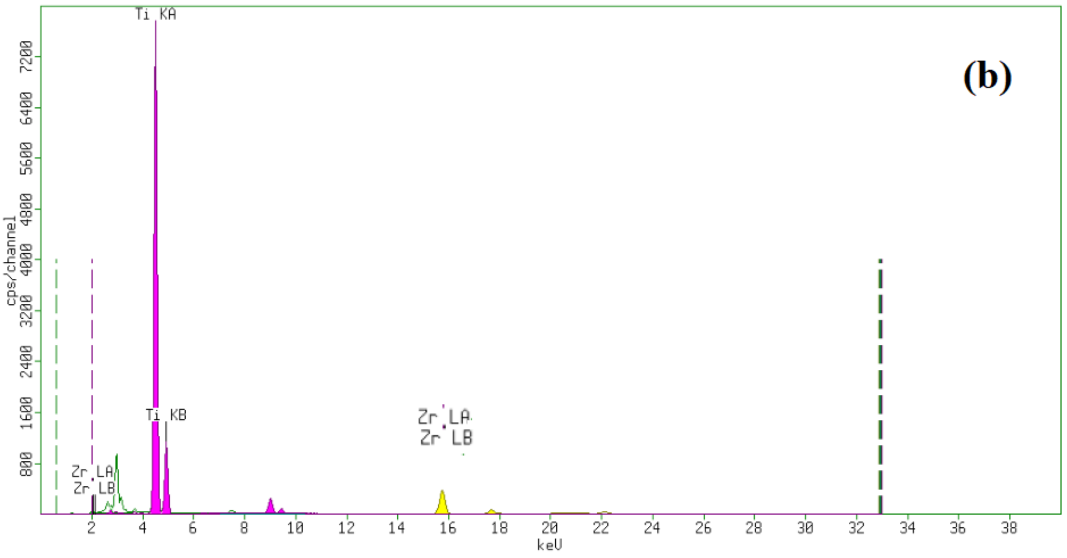

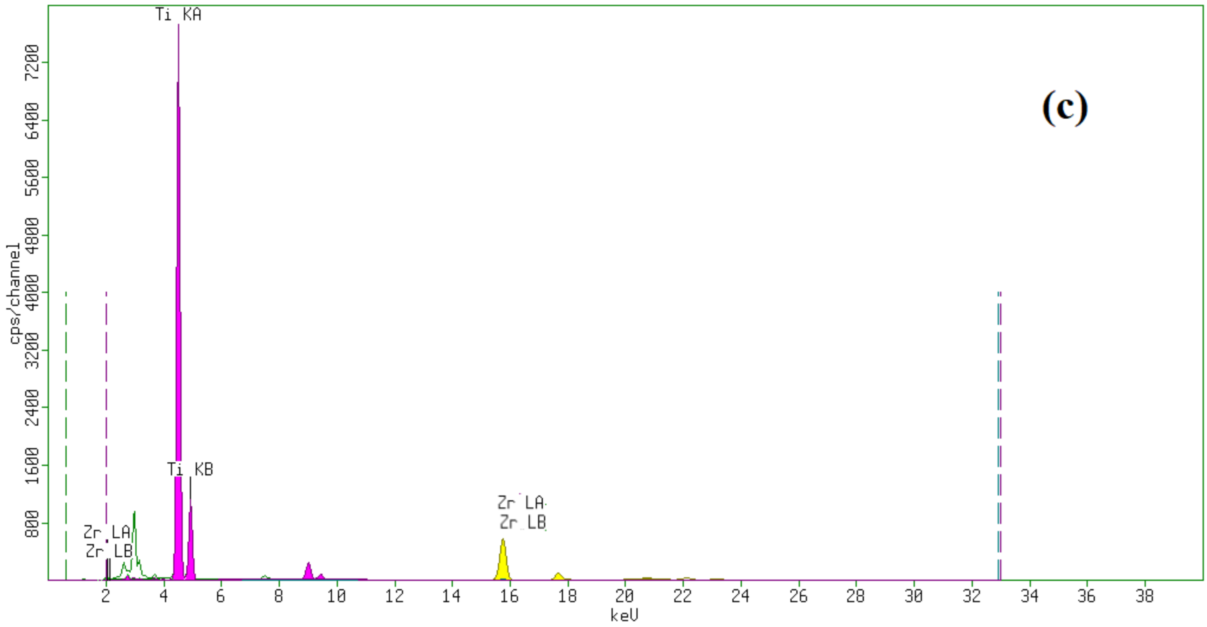
(c)
Ti KA
Ti KB
Zr LA
Zr LB
Zr LA
Zr LB
cps/channel
7200
6400
5600
4800
4000
3200
2400
1600
800
2
4
6
8
10
12
14
16
18
20
22
24
26
28
30
32
34
36
38
keV

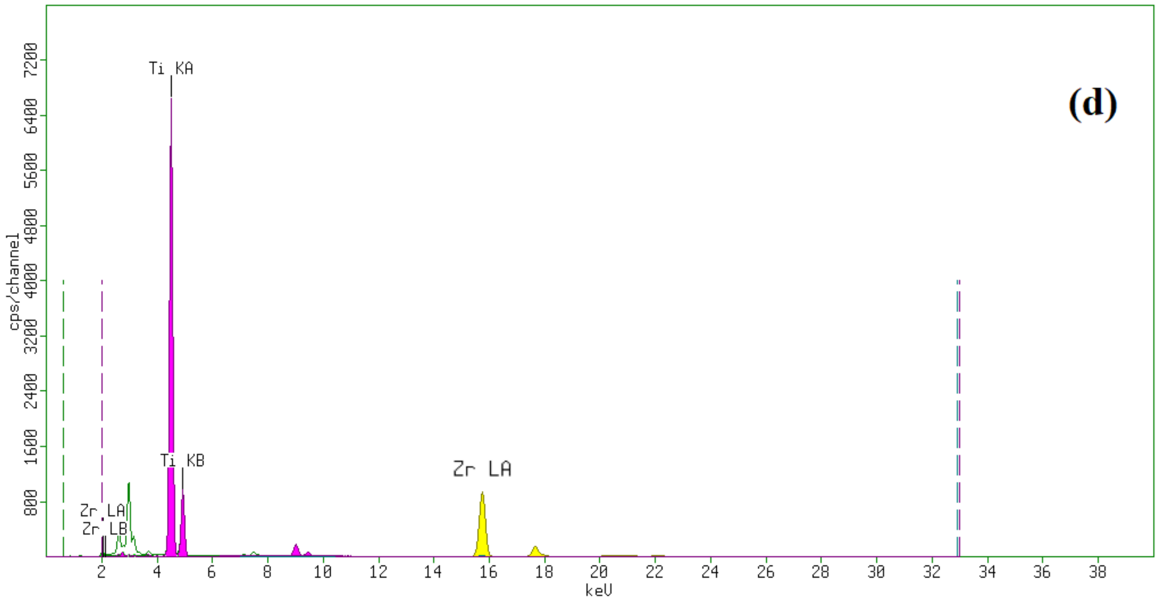
(d)
Ti KA
Ti KB
Zr LA
Zr LB
Zr LA
cps/channel
7200
6400
5600
4800
4000
3200
2400
1600
800
2
4
6
8
10
12
14
16
18
20
22
24
26
28
30
32
34
36
38
keV

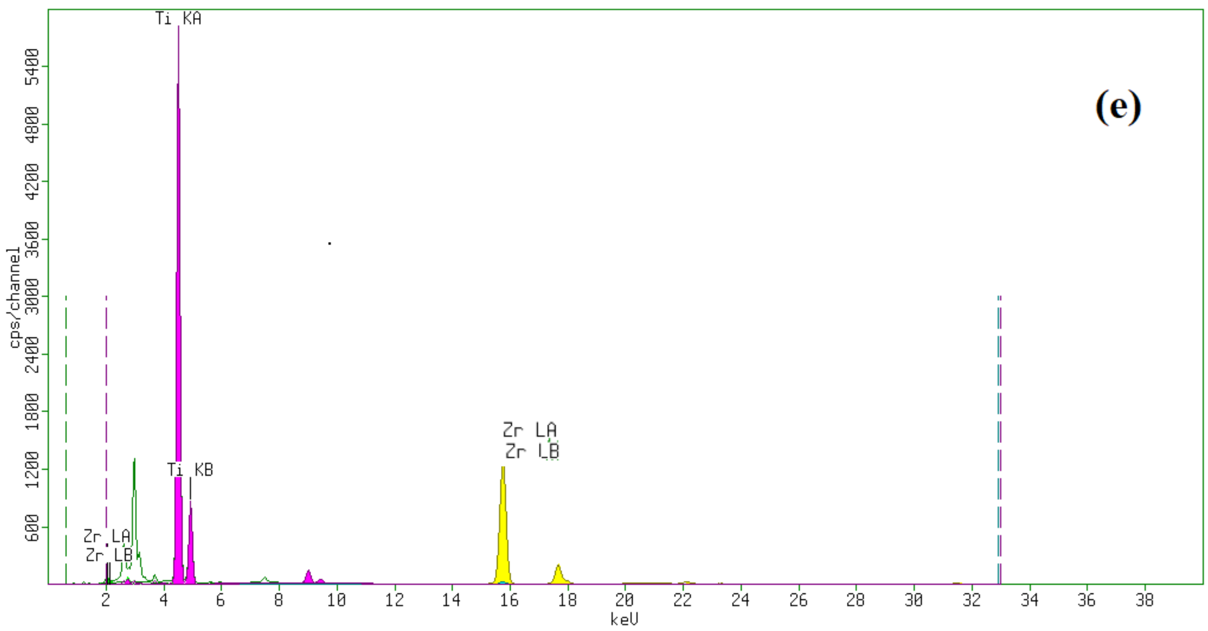

(e)
Ti KA
Ti KB
Zr LA
Zr LB
Zr LA
Zr LB
cps/channel
600
1200
1800
2400
3000
3600
4200
4800
5400
2
4
6
8
10
12
14
16
18
20
22
24
26
28
30
32
34
36
38
keV
